# Phrenic Nerve Transfer to Musculocutaneous Nerve: An Anatomical and Histological Study

**DOI:** 10.3390/life13091892

**Published:** 2023-09-11

**Authors:** Alexandra Fochtmann-Frana, Bettina Pretterklieber, Christine Radtke, Michael L. Pretterklieber

**Affiliations:** 1 Department of Plastic, Reconstructive and Aesthetic Surgery, Medical University of Vienna, Spitalgasse 23, 1090 Vienna, Austria; christine.radtke@meduniwien.ac.at; 2 Division of Macroscopic and Clinical Anatomy, Gottfried Schatz Research Center, Medical University of Graz, Auenbruggerplatz 25, 8036 Graz, Austria; bettina.pretterklieber@medunigraz.at (B.P.); michael.pretterklieber@medunigraz.at (M.L.P.); 3 Division of Anatomy, Center for Anatomy and Cell Biology, Medical University of Vienna, Waehringer Str. 13, 1090 Vienna, Austria

**Keywords:** phrenic nerve transfer, musculocutaneous nerve, Sudan black staining, supraclavicular approach, respiratory function, elbow flexor muscle function

## Abstract

Background: To restore elbow flexor muscle function in case of traumatic brachial plexus avulsion, the phrenic nerve transfer to the musculocutaneous nerve has become part of clinical practice. The nerve transfer can be done by means of video-assisted thoracic surgery without nerve graft or via supraclavicular approach in combination with an autograft. This study focuses on a detailed microscopic and macroscopic examination of the phrenic nerve. It will allow a better interpretation of existing clinical results and, thus, serve as a basis for future clinical studies. Material and Methods: An anatomical study was conducted on 28 body donors of Caucasian origin (female n = 14, male n = 14). A sliding caliper and measuring tape were used to measure the diameter and length of the nerves. Sudan black staining was performed on 15 µm thick cryostat sections mounted on glass slides and the number of axons was determined by the ImageJ counting tool. In 23 individuals, the phrenic nerve could be examined on both sides. In 5 individuals, however, only one side was examined. Thus, a total of 51 nerves were examined. Results: The mean length of the left phrenic nerves (33 cm (29–38 cm)) was significantly longer compared to the mean length of the right phrenic nerves (30 cm (24–33 cm)) (*p* < 0.001). Accessory phrenic nerves were present in 9 of 51 (18%) phrenic nerves. The mean number of phrenic nerves axons at the level of the first intercostal space in body donors with a right accessory phrenic nerve was significantly greater compared to the mean number of phrenic nerves axons at the same level in body donors without a right accessory phrenic nerve (3145 (range, 2688–3877) vs. 2278 (range, 1558–3276)), *p* = 0.034. A negative correlation was registered between age and the nerve number of axons in left (0.742, *p* < 0.001) and right (−0.273, *p* = 0.197) phrenic nerves. The mean distance from the upper edge of the ventral ramus of the fourth cervical spinal nerve to the point of entrance of the musculocutaneous nerve between the two parts of the coracobrachialis muscle was 19 cm (range, 15–24 cm) for the right and 20 cm (range, 15–25 cm) for the left arm. Conclusions: If an accessory phrenic nerve is available, it presumably should be spared. Thus, in that case, a supraclavicular approach in combination with a nerve graft would probably be of advantage.

## 1. Background

The phrenic nerve regularly receives fibers from the ventral rami of the segments C3, C4, and C5. However, it may also receive accessory fibers from the caudal cervical and even cranial thoracic segments. These additional rootlets can run independently for a varying length of time to form an accessory phrenic nerve. According to Lanz et al. [1], an accessory phrenic nerve is present in at least 10% of cases. This accessory phrenic nerve is formed in a large proportion (5% of cases) from the inferior trunk forming the brachial plexus (C8, Th1) [1,2]. The phrenic nerve is often described as a purely motor nerve, which is not true. Along its way the phrenic nerve receives afferent fibers from the pleura, the pericardium, and the peritoneum [1]. Furthermore, it contains proprioceptive fibers from the diaphragm and, thus, should be better classified as “muscular nerve”.

Patients with traumatic avulsion of the brachial plexus roots (C5—Th1) can profit from nerve transfer to the musculocutaneous nerve to restore elbow flexor muscle function [3,4,5]. Lurje et al. [5,6] were the first to suggest that the phrenic nerve may be used as a source of motor axons. In the last few decades, the phrenic nerve transfer to the musculocutaneous nerve has become part of clinical practice [3,4,5,7,8]. The qualitative systematic review by Cardoso et al. [9] showed that elbow flexor muscle strength had been graded ≥ M3 (movement against gravity is just possible) according to the British Medical Research Council scale [10,11] in 70.1% of patients following phrenic nerve transfer to the musculocutaneous nerve. Phrenic nerve transfer to the musculocutaneous nerve can be achieved by means of video-assisted thoracic surgery without nerve graft [3,5,9,12,13,14,15] or via supraclavicular approach in combination with an autograft [12,15,16]. It could be shown in a recent study that patients who underwent supraclavicular sectioning of the nerve in combination with an autograft had a significantly better clinical outcome (elbow flexion strength recovery) compared to patients treated by video-assisted thoracoscopic technique without nerve autograft [15]. Furthermore, Socolovsky et al. showed that the functional clinical outcome was better in patients with a shorter nerve graft than in patients with a longer nerve graft (9.8 vs. 15.1 cm) [9,16]. In order to explain these clinical results in a well-founded way, there is a lack of basic studies which deal in detail with the macroscopic and histological structure of the phrenic nerve. A possible reason for these clinical findings could be a different number of axons of the phrenic nerve at different heights.

In general, the phrenic nerve as donor is controversially discussed and some questions remain unanswered [9,17]. Former authors stated that the phrenic nerve used as donor is safe as patients usually did not develop respiratory symptoms postoperatively up to 10 years [9,18]. In spite of these results, there is a lack of long-term studies evaluating respiratory capacity in elderly patients following phrenic nerve transfer to the musculocutaneous nerve [17]. It is known from previous studies that the movement of the chest wall during respiration is not only due to the contraction of the diaphragm but is a complex movement involving many muscles [19]. However, the diaphragm is the most important player [20] in the respiratory process and can be weakened by many diseases or age-related phenomena (such as neuromuscular disorders, emphysema, obesity, or kyphosis) [21]. The sparing of an accessory phrenic nerve, which unites with the main nerve within the mediastinum, could possibly prove protective for the functional preservation of the diaphragm as respiratory muscle [1,2,21]. However, there are no data yet on the diameter and number of axons of the accessory phrenic nerve.

In the present work, four main objectives were to be elaborated:

(1) The main aim was to work out detailed knowledge on the macroscopic (length and diameter on different levels) and histological (number of axons) characteristics at different levels of the human phrenic nerve. It included intraindividual side comparison between left and right phrenic nerves taken from Caucasian male and female body donors;
(2) Furthermore, the presence of an accessory phrenic nerve was re-evaluated. If present, its macroscopic and histological characteristics have also been evaluated;
(3) An additional aim was to evaluate eventually age-dependent differences in the number of nerve fibers within the phrenic nerve;
(4) Moreover, the distance from the upper edge of the ventral ramus of the fourth cervical spinal nerve to the point of entrance of the musculocutaneous nerve between the two parts of the coracobrachialis muscle was measured. In addition, also macroscopically the diameters of the phrenic nerve, accessory phrenic nerve, and musculocutaneous nerve were measured.

## 2. Materials and Methods

### 2.1. Samples

This gross anatomical and histological study has been conducted on 28 body donors of Caucasian origin (female n = 14, male n = 14). The mean age of the deceased individuals was 82 years (range, 53–97 years). All experiments have been performed at the Division of Anatomy of the Medical University of Vienna, Austria. The bodies had been donated to medical education and research at our university. In addition to the informed consent of the deceased individuals, the study was approved by the ethics committee of our university (approval no. 1857/2020). The specimens have been fixed with a mixture of formalin and phenol, and underwent the regular dissection course for medical students. At the end of the course, we were able to examine the phrenic nerves on both sides in 23 individuals and in 5 specimens only unilateral (unilateral right: n = 2, unilateral left: n = 3). We included only specimens lacking any macroscopic signs of intrathoracic tumor diseases, deformities, or surgical scars.

### 2.2. Dissection, Measurement

The phrenic nerves were exposed by careful stratigraphic dissection. The length of the phrenic nerve from its commencement at the level of the ventral ramus of the fourth cervical spinal nerve as well as from the level of its passage dorsal to the cartilage of the first rib until its entrance into the diaphragm was determined by a measuring tape. In addition, the same method was used to measure the distance from the ventral ramus of the fourth cervical spinal nerve to the point of entrance of the musculocutaneous nerve between the two parts of the coracobrachialis muscle. A sliding caliper was used to measure the diameter of the phrenic, accessory phrenic, and musculocutaneous nerves. For further histological analysis, 1 cm long cross-sectional segments of the phrenic nerve were cut out at the level of the 1st intercostal space, and 1.5 cm cranial to the diaphragm. If an accessory phrenic nerve was present, a 1 cm cross-sectional segment was also harvested centrally.

### 2.3. Tissue Preparation, Staining, and Histological Analysis

After harvesting the nerve cross-sections, they were stored in 4% formalin (ROTI^®^ Histofix 4%, Carl Roth GmbH, Karlsruhe, Germany). After washing in phosphate buffered saline (PBS) overnight at 4 °C, the nerve samples were dehydrated in increasing sucrose/distilled water solutions (10%, 20%, and 40%). The probes were embedded using Tissue-Tek^®^ O.C.T.^™^ (Sakura Finetek Europe B.V., Zoeterwoude, The Netherlands) and shock-frozen at −80 °C in 2-methyl-butane. 15 µm thick cryostat-sections were obtained and mounted on SuperFrost Ultra Plus^©^ slides (Thermo Fisher Scientific/Menzel-Gläser, Braunschweig, Germany). The slides were dried at room temperature for 30–60 min and subsequently stored for 30–60 min in distilled water. After washing the slides in 50% ethanol for 1–3 min they were stained with Sudan black B staining solution for 5 min and subsequently washed in distilled water. The slides were covered using Kaiser’s glycerol gelatine (Carl Roth GmbH, Germany).

**Figure 1:**
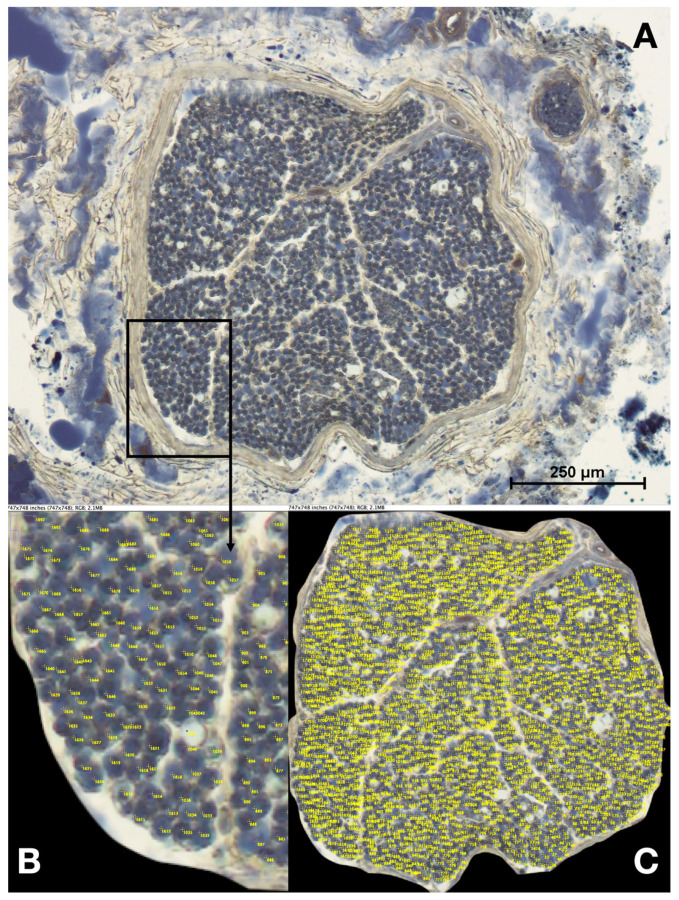
Evaluating the number of axons using ImageJ. (**A**) Overview of a segment taken from a right phrenic nerve 1.5 cm cranial to the diaphragm, female individual, who died at the age of 79 years. (**B**) Detail view of the same specimen to show the method of counting axons using the ImageJ counting tool. (**C**) Overview of the finished counting revealing at total number of 1914 axons.

The slides were analyzed using a light microscope (Nikon Eclipse 80i, Nikon Corp., Tokyo, Japan) and photographed with the corresponding camera (Nikon Coolpix 995, Nikon Corp., Tokyo, Japan). The number of axons was determined by the ImageJ counting tool (ImageJ version 1.53a, Wayne Rasband, National Institutes of Health, Bethesda, MD, USA) (see Figure 1).

### 2.4. Statistical Analysis

Continuous data were described as means, minimum values, and maximum values. T-tests were used to compare the mean values of two groups. Nominally or ordinally scaled values were compared using the chi-square test. Spearman’s rank correlation coefficient was calculated to determine a possible correlation between age and number of axons. A two-sided *p*-value < 0.05 was considered significant. Statistical analysis was performed using the Statistical Package for Social Sciences software (SPSS version 26.0; IBM SPSS Statistics, Armonk, NY, USA).

## 3. Results

In 5 individuals, the phrenic nerve could only be investigated on one side (right: n = 2, left: n = 3). In the remaining 23 individuals, both sides could be evaluated. Thus, 51 phrenic nerves were investigated.

The mean length of the left phrenic nerve (33 cm, range 29–38) was significantly longer than the length of the right phrenic nerve (30 cm, range (24–33), *p* < 0.001) (see Table 1). In addition, an intraindividual comparison of right and left phrenic nerve was performed. In only two (9%) specimens, both phrenic nerves were of the same length. In 5 (22%) individuals, the left phrenic nerve was 2 cm longer than the right phrenic nerve. In 2 (9%) specimens, the left phrenic nerve was 3 cm longer, in 6 (26%), 4 cm longer, in 6 (26%), 5 cm longer, in 1 (4%), 7 cm, and in another 1 (4%), 8 cm longer than the right phrenic nerve. Intraindividual comparison of the 23 individuals with bilateral phrenic nerves showed that the left phrenic nerve was, on average, 3.7 cm (range 0–8 cm) longer than the right phrenic nerve.

On the right side of the body, the mean distance from the upper edge of the ventral ramus of the fourth cervical spinal nerve to the point of entrance of the musculocutaneous nerve between the two parts of the coracobrachialis muscle was 19 cm (range, 15–24 cm), and on the left side of the body the mean of this distance was 20 cm (range, 15–25 cm). The mean diameter of the left musculocutaneous nerve measured at its entrance into the coracobrachialis muscle was significantly greater compared to the mean diameter of the left phrenic nerve (3.4 mm (range, 2.2–4.4 mm) vs. 1.8 mm (range, 1.1–2.5 mm) *p* < 0.001). Similarly, the mean diameter of the right musculocutaneous nerve was significantly greater compared to the mean diameter of the right phrenic nerve (3.5 mm (range, 2–4.8 mm) vs. 1.8 mm (range, 1–2.7 mm) *p* < 0.001). Details on diameter and length of the phrenic nerve are listed in Table 1.

**Figure 2:**
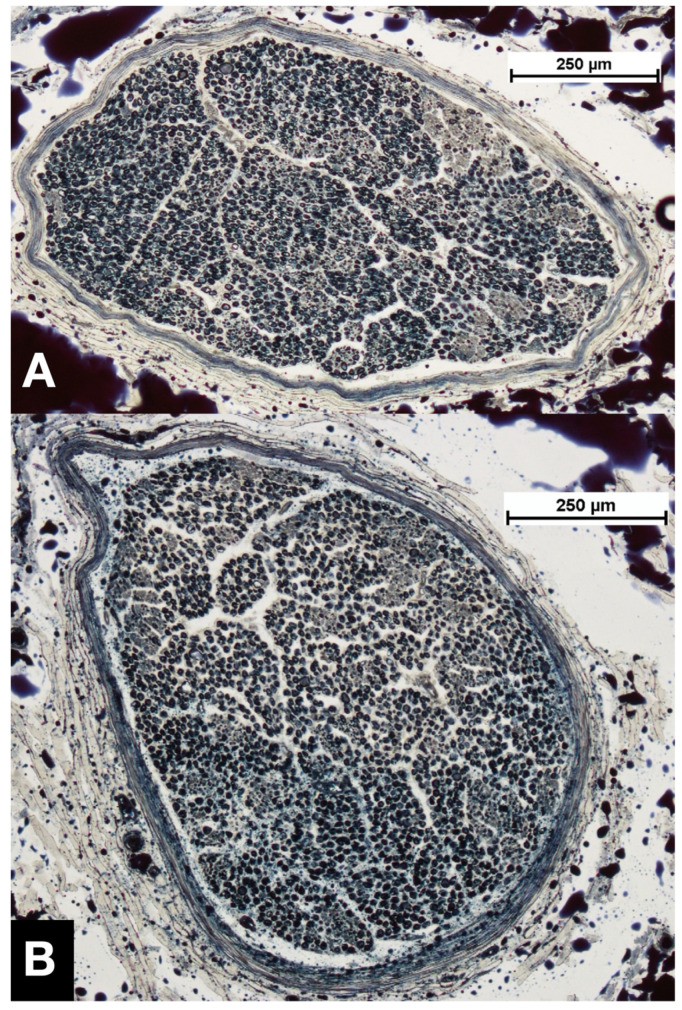
Comparison of phrenic nerve segments taken from one individual. (**A**) Overview of a segment from the right phrenic nerve taken at the level of the 1st intercostal space, 2517 nerve fibers. (**B**) Overview of a segment from the right phrenic nerve excised 1.5 cm cranial to the diaphragm, 2298 axons. The specimens were taken from a male individual, age at death 86 years.

**Figure 3:**
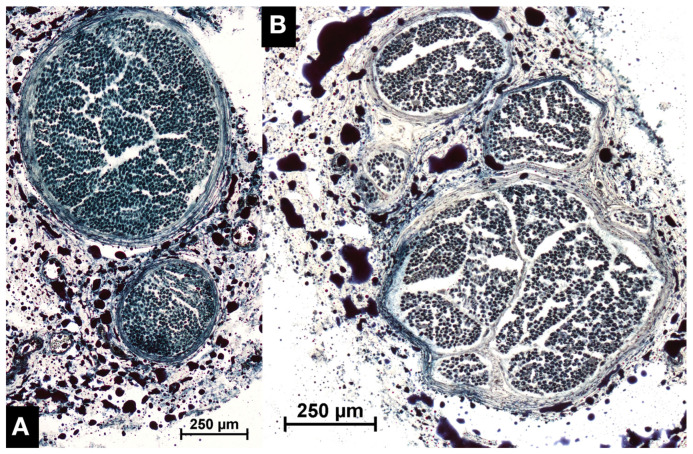
Comparison of phrenic nerve segments taken 1.5 cm cranial to the diaphragm. (**A**) Male individual, age at death 88 years, 2384 nerve fibers. (**B**) Male individual, age at death 91 years, 2159 axons.

In both sexes, the mean number of nerve fibers of phrenic nerve cross-sectional segments taken at the level of the 1st intercostal space was higher compared to that taken 1.5 cm cranial to the diaphragm (Figure 2 and Figure 3, Table 2). However, in 6 (21%) individuals the difference in number of axons presented in the opposite direction: in a 97-year-old female individual, 1.5 cm cranial to the diaphragm the left phrenic nerve contained 1871 nerve fibers more than at the level of the 1st intercostal space. In this specimen, an accessory phrenic nerve consisting of 1440 nerve fibers fused with the phrenic nerve caudally to the 1st intercostal space. Nevertheless, the total number 1.5 cm cranial to the diaphragm was higher than to be expected by simply adding the number of nerve fibers of the phrenic nerve at the level of the 1st intercostal space and the accessory phrenic nerve. The remaining 5 individuals with a greater number of nerve fibers 1.5 cm cranial to the diaphragm compared to the level of the 1st intercostal space lacked an accessory phrenic nerve. In a 92-year-old woman, both phrenic nerves contained more nerve fibers (right +368 and left +176) at the level close to the diaphragm than at the level of the 1st intercostal space. Furthermore, the right phrenic nerve of a 93-year-old female body as well as of an 88-year-old male body donor and a 96-year-old female body donor each showed a surplus of 93, 54, or even 317 nerve fibers, respectively. And, finally, the left phrenic nerve of a 91-year-old male body donor presented with a surplus of 415 nerve fibers.

Accessory phrenic nerves were present in 9 of 51 (18%) phrenic nerves. Bilateral accessory phrenic nerves occurred in 2 cases, a right unilateral accessory phrenic nerve also in 2, and a left unilateral accessory phrenic nerve in 3 individuals (see Table 3). The mean number of nerve fibers of the right phrenic nerve at the level of the 1st intercostal space in individuals presenting a right accessory phrenic nerve was significantly greater compared to that in specimens lacking a right accessory phrenic nerve (3145 (range, 2688–3877) vs. 2278 (range, 1558–3276), *p* = 0.034, see Table 4). At this point, it should be noted that in all cases, the accessory phrenic nerve united with the main nerve distal to the measurement site.

Sex-specific differences concerning the number of axons are summarized in Table 5. On the right body side, the mean number of axons of the cross-sectional segments taken from female body doners 1.5 cm cranial to the diaphragm was higher compared to the mean number of axons of the cross-sectional segments taken from male body doners at the same height (2396 (range, 1423–3190) vs. 2036 (range, 1539–2430), *p* = 0.081, see Table 5).

**Figure 4:**
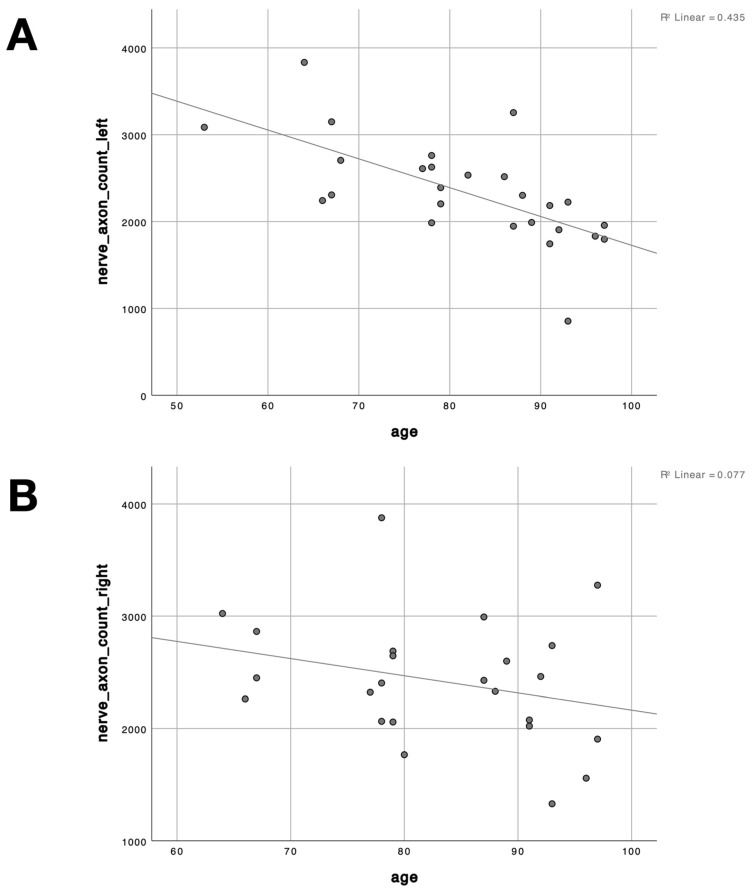
Negative correlation between age and the number of axons. (**A**) negative correlation between age and the number of axons in left phrenic nerves (Spearman’s correlation coefficient −0.742, *p* < 0.001). (**B**) negative correlation between age and the number of axons in right phrenic nerves (Spearman’s correlation coefficient −0.273, *p* = 0.197).

A negative correlation was registered between age and number of axons in left (Spearman’s correlation coefficient −0.742, *p* < 0.001) and right (Spearman’s correlation coefficient −0.273, *p* = 0.197) phrenic nerves (see Figure 4).

## 4. Discussion

The phrenic nerve is a tempting donor to restore elbow flexor muscle function in patients with brachial plexus injuries, thus avoiding any free nerve graft. However, as revealed by the present results, the left and right phrenic nerves significantly differ in their overall length. Thus, the left phrenic nerve turned out to be significantly longer than the right one. Similar results have been presented by Jiang et al. [22,23], as in their specimens also the left phrenic nerve was—on average—six centimeters longer than its right counterpart. This finding is sufficiently explained by the elevation of the diaphragm caused by the right-sided liver and due to the left position of the heart and the pericardium. Hence, the left phrenic nerve must follow an arc, whereas the right phrenic nerve is able to descend in an almost vertical direction to reach the diaphragm [22,24].

If the anterior division of the upper trunk is unaffected, a direct nerve transfer of the phrenic nerve via supraclavicular approach to the anterior division of the upper trunk could be performed. However, this procedure may be associated with axonal misrouting caused by choosing a too proximal target [15]. Thus, a direct phrenic nerve transfer to the musculocutaneous nerve is certainly preferable. For this reason, we focus in the present work on the phrenic nerve transfer to the musculocutaneous nerve. Phrenic nerve transfer to the musculocutaneous nerve can be performed by means of video-assisted thoracic surgery without nerve graft [3,5,9,12,13,14,15] or via supraclavicular approach in combination with an autograft [12,15,16]. One would expect that the clinical outcomes of patients treated with video-assisted thoracic surgery without nerve graft would be better than the clinical outcomes of patients treated with supraclavicular approach in combination with an autograft. However, it is exactly the other way around. It could be shown in a recent study that patients who underwent supraclavicular sectioning of the nerve in combination with an autograft had a significant better clinical outcome (elbow flexion strength recovery) compared to patients treated by video-assisted thoracoscopic technique without nerve autograft [15]. Since there is no sufficient explanation for these clinical findings, it seems reasonable to perform a more detailed microscopic and histological examination of the phrenic nerve.

### 4.1. Consequence of the Frequently Present Accessory Phrenic Nerve

Lanz et al. [1] described that an accessory phrenic nerve is present in at least 10% of the population. Other authors state that an accessory phrenic nerve occurred in one-third of their population [25]. In the present study, accessory phrenic nerves were present in nine of 51 (18%) phrenic nerves. The frequency of occurrence is, thus, midway between previous studies. However, various views exist in the existing literature regarding the definition of an accessory phrenic nerve. For example, Loukas et al. [26] counts all nerves contributing to the phrenic nerve after crossing the anterior scalene as accessory phrenic nerves. He even included the supraclavicular nerves which, apparently, course far too superficially to get in direct contact with the phrenic nerve. This makes objective quantification difficult based on the available literature. We adhere to the classic definition of accessory phrenic nerve, as described in the background section of this paper. Assuming that an accessory phrenic nerve is present in 10–30% of the population, it may be clinically advantageous to determine the possible presence of an accessory phrenic nerve by radiologic imaging in patients who are scheduled for a phrenic nerve transfer. However, radiological imaging of the accessory phrenic nerve seems to be still a theory. At present, it is not yet possible to image the accessory phrenic nerve even with high-resolution ultrasound or MR imaging. Thus, further studies on that topic are apparently necessary (Bodner, G. (2023) personal communication). Furthermore, the morbidity of lifting during a phrenic nerve transfer is not negligible. Luedemann et al. [17] found a significant reduction of vital capacity after full-length right phrenic nerve transfer. Therefore, constant consideration should be given to how to cause the least functional loss to the affected patient and still achieve a good clinical outcome. Thus, the presence of an accessory phrenic nerve could be used as an advantage. In the current study, in 67% of the phrenic nerves which were accompanied by an accessory phrenic nerve, the accessory phrenic nerve did not join the phrenic nerve until caudal to the 1st intercostal space. Hypothetically, it would be advantageous in these individuals not to lift the phrenic nerve in its full length but to use the supraclavicular approach in combination with an autograft. A certain continuity of the main nerve would be preserved and, possibly, less lifting morbidity would occur. The usefulness of this hypothesis depends, of course, on the fiber distribution and the overall number of axons of the accessory phrenic nerve. The main question would be how many motor axons join the accessory phrenic nerve? There are currently no data on this topic.

Furthermore, in the current study, we were able to show that the mean number of axons at the level of the 1st intercostal space in body donors with a right accessory phrenic nerve was significantly greater compared to the mean nerve number of axons at the same level in body donors without a right accessory phrenic nerve. This result is rather unexpected. With regard to the median age, the groups do not differ. A higher age in the group without accessory phrenic nerve could have served as an explanation for this result. However, this was not the case. The level at which the accessory phrenic nerve joins the main nerve is described in Table 3.

### 4.2. Value of Counting the Axons

As was to be expected, in the present study we were able to show that the mean number of axons of the phrenic nerve at the 1st intercostal space was greater compared to number of axons 1.5 cm cranial to the diaphragm. However, in 6 individuals, the difference in the number of axons presented antithetically. A possible explanation could be the presence of an accessory phrenic nerve that fuses more caudally within the mediastinum with the phrenic nerve (Table 3). As a direct consequence the nerve fibers from the accessory phrenic nerve are not included in the number of axons at the level of the 1st intercostal space. Thus, the number of axons at the level of the diaphragm is higher compared with the level of the 1st intercostal space. In the present study this was the case in 1 body donor. For the remaining 5 individuals, an explanation would be the presence of accessory branches joining the phrenic nerve intrathoracally. It has been shown that such branches can arise from the ventral branch of the first to third cervical spinal nerves or from the sympathetic trunk (via grey rami communicantes), which may even reach the diaphragm entirely independent of the phrenic nerve [1,2]. This phenomenon was first described by Felix et al. in 1922 [17,27]. The presence of intrathoracic accessory would strengthen the use of a supraclavicular approach in combination with an autograft to restore elbow flexor muscle function and maintain a residual function of the nerve at the affected side, even in patients without accessory phrenic nerve.

### 4.3. Influence of Age

It was previously shown that older persons, in general, are prone to a higher risk of developing respiratory impairment following phrenic nerve transfer [9,28,29,30]. There is a lack of long-term studies evaluating pulmonary function in elderly patients following transfer of the phrenic to the musculocutaneous nerve. We were able to show a negative correlation between age and the number of axons in both phrenic nerves. This finding indicates that the reduction of respiratory capacity in the elderly is not merely due to a loss of intercostal muscle mass and calcification of costal cartilages [9,30] but also to a reduction of functional axons in the phrenic nerve. The correlation between the number of axons and age reached the significance level only on the left side. Of course, the limitations of our work must be mentioned at this point. A possible explanation for this result could be a too small sample size. Possibly, the significance level would have been reached with a larger number of cases. Of course, with the present study we can only describe tendencies and cannot give clinical therapy recommendations. However, there are indications in our data that a phrenic nerve transfer in older patients is not as effective as in younger patients. It seems that nerve regeneration may not be as efficient with a reduced number of axons as with a higher number of axons in the donor nerve. In addition, the functional deficits (such as respiratory impairment) associated with this type of nerve transfer must always be taken into account.

## 5. Conclusions

Although it is actually not yet possible to visualize an accessory phrenic nerve by radiological imaging, we can assume that 10–30% of the population have an accessory phrenic nerve. Thus, the supraclavicular approach in combination with a nerve graft is the better surgical option to preserve a possible accessory phrenic nerve. In addition, a partial continuity of the phrenic nerve would be preserved in a considerable proportion of the population. Of course, this only applies to patients in whom the accessory phrenic nerve is present and in whom it fuses with the phrenic nerve caudally to the clavicle. However, there is a need to confirm this hypothesis in a clinical study.

## Figures and Tables

**Table 1:** Side-specific differences regarding diameter and length of the phrenic nerves.

	Phrenic Nerve R	Phrenic Nerve L	*p*
diameter clavicle ^1^ (mean, range in mm)	1.6 (1–2.9)	1.6 (1–2.9)	0.895
diameter ICS ^2^ (mean, range in mm)	1.6 (1–2.7)	1.6 (1–2.9)	0.886
diameter 10cm ^3^ (mean, range in mm)	1.8 (1–2.5)	1.9 (1–2.6)	0.208
diameter diaphragm ^4^ (mean, range in mm)	2.1 (1–2.9)	1.9 (1.2–2.7)	0.106
length C4 ^5^ (cm)	30 (24–33)	33 (29–38)	<0.001
length clavicle ^6^ (cm)	23 (18–27)	26 (23–39)	<0.001

^1^ diameter of the phrenic nerve measured at the level of the clavicle; ^2^ 1st intercostal space; ^3^ measured 10 cm from the 1st intercostal space; ^4^ measured 1.5 cm cranial to the diaphragm; ^5^ length measured form C4; ^6^ length measured form upper edge of the clavicle.

**Table 2:** Effects of sidedness and sexual dimorphism in the number of axons of both phrenic nerves.

Individuals (n = 28) ^1^	Age (Mean, Range)	Phrenic Nerve R (*n*)	Phrenic Nerve L (*n*)	Number of Axons_ICS ^2^ R (Mean, Range)	Number of Axons_ICS ^2^ L (Mean, Range)	*p*	Number of Axons_ Diaphragm ^3^ R (Mean, Range)	Number of Axons_ Diaphragm ^3^ L (Mean, Range)	*p*
Male (n = 14)	80 (53–91)	11	13	2301 (1767–2646)	2341 (1744–3086)	0.755	2036 (1539–2430)	2140 (1315–2886)	0.477
Female (n = 14)	83 (67–97)	13	14	2526 (1330–3877)	2337 (3833–854)	0.509	2396 (1423–3190)	2087 (858–3668)	0.247

^1^ phrenic nerve: double-sided (n = 23), phrenic nerve: unilateral right (n = 2), phrenic nerve: unilateral left (n = 3); ^2^ phrenic nerve cross-sectional segment 1st intercostal space; ^3^ phrenic nerve cross-sectional segment 1.5 cm cranial to the diaphragm.

**Table 3:** Incidence of the accessory phrenic nerve.

	Number of Axons R ^3^ (Mean, Range)	Number of Axons L ^3^ (Mean, Range)	Length R ^3^ (cm)	Length L ^3^ (cm)	Diameter R ^3^ (mm)	Diameter L ^3^ (mm)	Anatomic Information R ^3^	Anatomic Information L ^3^
bilateral, f ^1^, 78 ^2^	775	518	12	11	1	0.9	derived from C5, joined phrenic nerve in the thorax	derived from C5, joined phrenic nerve in the thorax
bilateral, f ^1^, 64 ^2^	1540	1313	10	5	1	1	derived from C5, joined phrenic nerve at the level of the 1st intercostal space	derived from C5, joined phrenic nerve on the level of the subclavian vein
unilateral, R ^3^, f ^1^, 79 ^2^	889		5		0.9		derived from C5, joined phrenic nerve in the thorax	
unilateral, R ^3^, f ^1^, 87 ^2^	890		7.5		0.6		derived bipartitely from C5, joined phrenic nerve in the 1st intercostal space	
unilateral, L ^3^, f ^1^, 97 ^2^		1440		15		1.1		derived bipartitely from C5, joined phrenic nerve in the thorax
unilateral, L ^3^, m ^1^, 91 ^2^		502		15		1.1		derived from C5, joined phrenic nerve in the thorax
unilateral, L ^3^, m ^1^, 66 ^2^		1336		12		1.3		derived from C5, joined phrenic nerve in the thorax

^1^ sex (f = female, m = male); ^2^ age (in years); ^3^ anatomic side (R = right, L = left).

**Table 4:** Nerve number of axons at the level of the 1st intercostal space in body donors with accessory phrenic nerve and body donors without accessory phrenic nerve.

	Body Donors with APN ^2^	Body Donors without APN ^2^	*p*
number of axons_ICS ^1^ R (mean, range)	3145 (2688–3877)	2278 (1558–3276)	0.034
number of axons_ICS ^1^ L (mean, range)	2449 (1744–3833)	2314 (854–3255)	0.749

^1^ phrenic nerve cross-sectional segment 1st intercostal space; ^2^ accessory phrenic nerve.

**Table 5:** Effects of sexual dimorphism in the number of axons of both phrenic nerves.

Individuals (n = 28) ^1^	Male (n = 14)	Female (n = 14)	*p*
number of axons_ICS ^2^ R (mean, range)	2301 (1767–2646)	2526 (1330–3877)	0.31
number of axons_ICS ^2^ L (mean, range)	2341 (1744–3086)	2337 (3833–854)	0.987
number of axons_diaphragm ^3^ R (mean, range)	2036 (1539–2430)	2396 (1423–3190)	0.081
number of axons_diaphragm ^3^ L (mean, range)	2140 (1315–2886)	2087 (858–3668)	0.817

^1^ phrenic nerve: double-sided (n = 23), phrenic nerve: unilateral right (n = 2), phrenic nerve: unilateral left (n = 3); ^2^ phrenic nerve cross-sectional segment 1st intercostal space; ^3^ phrenic nerve cross-sectional segment 1.5 cm cranial to the diaphragm.

## Data Availability

Not applicable.
